# Cost-effectiveness and clinical impact of robotic-assisted hepatectomy

**DOI:** 10.1007/s11701-025-02319-z

**Published:** 2025-04-14

**Authors:** A. Rouault, F. Pecquenard, M. Elamrani, E. Boleslawski, S. Truant, G. Millet

**Affiliations:** Lille University Hospital, Lille, France

**Keywords:** Cost-effectiveness, Hepatectomy, Robot

## Abstract

Robotic-assisted hepatectomy has gained traction in hepatobiliary surgery, but its cost-effectiveness compared to traditional surgical approaches remains unclear. This study investigates clinical outcomes and financial implications of robotic-assisted liver surgery in a high-volume center, comparing it with open and laparoscopic methods. A retrospective cohort study was conducted on patients undergoing hepatectomy at Lille University Hospital in 2018 and 2021, performed by the institution’s first experienced robotic hepatobiliary surgeon. Data on patient demographics, intraoperative details, postoperative outcomes, and costs were analyzed. Costs included hospital stays, surgical materials, and complications, derived from national cost studies. A total of 111 patients were included, with a rise in minimally invasive procedures from 47.5% in 2018 to 75% in 2021. Robotic-assisted hepatectomy was associated with reduced hospital stays, lower complication rates, and fewer severe morbidities compared to laparotomy. The average cost per procedure (all surgical approaches combined) decreased from €12,169 in 2018 to €8,513 in 2021, with robotic surgery offering a significant financial advantage. The total savings for the 71 patients in the 2021 cohort was €259,576, driven primarily by reduced hospitalization times and fewer complications. Robotic-assisted hepatectomy is clinically safe and cost-effective, offering substantial financial savings over traditional surgery. The reduction in postoperative complications and hospital stay durations, particularly for complex cases, highlights the advantages of robotic surgery in hepatobiliary procedures. As surgical expertise increases, robotic surgery represents a sustainable and efficient alternative in liver resection.

## Introduction

Hepatobiliary surgery in France has seen a steady increase in procedures. The French National Hospital Discharge Database (Programme de Médicalisation des Systèmes d’Information, PMSI), which gathers data from all public and private French hospitals, indicates a 2% rise in hepatectomies between 2007 and 2012, reaching 14 per 100,000 inhabitants, or roughly 7500 procedures per year [1]. In France, the yearly incidence of minimally invasive liver surgery doubled from 16.5% in 2013 to 35.4% in 2022 [2].

Although the advantages of minimally invasive liver resection have already been documented, particularly in cirrhotic patients, the adoption of this surgical technique has been slower compared to other surgical fields [3]. One major reason is the technical difficulty of minimally invasive hepatectomy, which requires advanced skills in dissection, suturing, exposure, liver mobilization, and parenchymotomy. Robotic technology offers a tool to overcome certain difficulties.

The development of robotic surgery in digestive surgery is tied to the Da Vinci system by Intuitive Surgical Inc., Sunnyvale, California, USA. The Da Vinci Standard System, first introduced in 1998, has evolved into the Xi model with four interchangeable arms and seven degrees of freedom [4].

A 2020 report by the French National Academy of Surgery [5] highlighted a significant global increase in robotic surgery across all disciplines. In 2012, around 425,000 procedures were performed using robotic systems. By 2019, this number exceeded 1 million, with 5000 robots in use worldwide, including 134 in France. In 2022, 6.2% of the 44,058 general and digestive surgery hospital stays (excluding bariatric surgery) in France involved robot-assisted procedures.

A 2018 consensus statement [6] provided seven recommendations for robotic surgery in hepatobiliary fields, indicating that both major and minor hepatectomies with robotic approaches are feasible and safe, with similar oncologic outcomes to open surgery. The consensus also suggested comparable results between robotic and laparoscopic surgeries in hepatobiliary contexts. These recommendations have broadened surgical indications supported by technical advancements [7–9]. The feasibility and the safety of posterior hepatectomies with robotic surgery have been particularly noteworthy, achieving results difficult to obtain with a laparoscopic approach [10].

Despite growing acceptance, cost concerns persist among surgical teams. Literature analysis is limited due to study heterogeneity and training biases. Additionally, the length of hospital stays must be considered to balance the expenses of direct operative and postoperative costs [11].

Cost studies in other disciplines often compare open surgery with robotic surgery, finding advantages to robotics due to shortened hospital stays [12, 13]. We propose conducting a cost study on liver surgery to demonstrate the cost-effectiveness of robotic surgery in the comprehensive management of patients by an experienced hepatobiliary surgeon in the robotic field.

## Methods

This study was conducted in the Digestive Surgery and Transplantation Department of the Lille University Hospital. After obtaining approval from the institutional review board, a retrospective evaluation was conducted on patients who had undergone hepatectomies. Inclusion criteria were all hepatectomies in 2018 and 2021 performed by the first HPB robotic surgeon of the institution. Minor patients and cases with intraoperative discovery of unresectable tumors were excluded from the study.

Preoperative and intraoperative data were derived from a larger prospectively designed registered multicenter observational study investigating the objective and subjective determinants of the postoperative course of hepatectomy. This study was deemed non-interventional by the ethical committees of participating institutions and approved by the Data Protection Authority and Health Information Protection Committee (CNIL). All patients were informed that data are collected in a prospectively maintained database and analyzed retrospectively for this study (MR 004 declaration at CNIL).

The etiological diagnosis and the management were validated during a multidisciplinary consultation meeting (RCP) involving medical and surgical specialists. Preoperative data collected included gender, age, BMI, and WHO score. Preoperative liver function was evaluated using the CHILD–PUGH classification [14]. A MELD score [15] including total bilirubin, INR, and creatinine was also calculated. The difficulty in hepatectomies was evaluated using the IWATE score [16, 17]. All hepatectomies were performed in a high-volume hepatobiliary center by a surgeon with prior experience of more than 350 hepatectomies (open and minimally invasive). Surgical resections were performed according to RCP recommendations using open, laparoscopic, or robot-assisted approaches with the Da Vinci® Xi robot (Intuitive Surgical SARL). Liver segments were identified based on Couinaud’s segmental anatomic classification [18]. Anatomic resections were defined based on the Brisbane 2000 Terminology of Liver Anatomy and Resections [19]. Major liver resections were defined by resection of three or more adjoining Couinaud segments. A segmentectomy was defined as the complete resection of an area supplied by a third-order branch of the portal vein.

During the perioperative and postoperative phases, duration of surgery, need for transfusion, and lengths of stay in conventional care, intensive postoperative care, and continuous care were analyzed. Post-operative morbidity was assessed according to Clavien–Dindo [20]. A postoperative complication of grade 3 or more was considered severe.

### Cost evaluation

Direct costs were analyzed in euros (€). The cost study is derived from the 2019 national cost study (Étude Nationale des Coûts) [21], the 2019 work unit cost benchmark (Référentiel du RTC) [22], and the example of calculating the operating cost of an operating theater with a post-interventional monitoring room [23].

The duration of surgery was determined for each patient. Median duration of surgery combined with the cost of the operating room per minute (10.80€ [23]) gave the cost of the procedure for each surgical approach.

Mean length of stay (LOS) was extracted from our database and calculated based on the daily cost of each unit where the patient was hospitalized (conventional care: €491, intensive care unit: €2069, and critical care unit: €1472), as well as the length of stay in each of these units. The first night before surgery was excluded from the LOS to avoid bias as some patients were admitted the day before surgery, while others were admitted on the morning of the surgery, depending on their position in the surgical schedule. Data on severity were extracted from the French PMSI (Program for the Medicalization of Information Systems). Patients within the same GHM (Homogeneous Group of Patients) were classified from I to IV based on their postoperative complications and comorbidities. Patients in groups I and II were considered standard, and the cost per patient was calculated by multiplying the LOS in each unit by the daily cost of that unit. Patients in groups III and IV, who experienced postoperative complications, had their costs adjusted according to the French national cost study [21]. The additional cost for complications is calculated as follows:

$${\text{Cost in conventional unit}}: \left[ {{\text{LOS }}\left( {{\text{III}}{-}{\text{IV}}} \right) \, {-}{\text{ LOS }}\left( {{\text{I}}{-}{\text{II}}} \right)} \right] \, \times {\text{ cost 15}}0\% \, \times \, \% {\text{complicated patients }} \times \, 0.{85}$$

$${\text{Cost in ICU}}: \, \left[ {{\text{LOS ICU }}\left( {{\text{III}}{-}{\text{IV}}} \right) \, {-}{\text{ LOS ICU }}\left( {{\text{I}}{-}{\text{II}}} \right)} \right] \times {\text{ cost ICU }} \times \, \% {\text{complicated patients }} \times \, 0.{85}$$

Therefore, patients with severity index I–II defined the standardized cost in our institution, while the cost was adjusted for patients with severity index III–IV, considering that complications explain 85% of the cost and pre-operative comorbidities 15% [21].

For all techniques (laparotomy, laparoscopy, and robot-assisted), perioperative costs were evaluated using the individual costs of all disposable and partially reusable materials. The costs of robot-assisted materials include instrumentation (forceps, bipolar, vessel sealer, stapler, clip applicator) and specific disposable materials such as robotic drapes. One-year global costs were used to estimate the mean per-procedure cost. Costs associated with depreciation and maintenance of the robotic platform were not included, nor were the costs of hardware systems (e.g., CUSA, endoscope, generator). For each surgery, we defined a reference instrument: the CUSA® Excel + ultrasonic dissector for the laparotomy approach, Medtronic Ligasure for the laparoscopic approach, and Vessel Sealer for the robotic approach. Prices include all taxes.

In addition to evaluating the cost of disposable materials, fixed costs associated with the Da Vinci Xi system were also assessed. The operating cost of the robot was calculated by factoring in the initial investment (including the robotic system, additional optics, and specialized sterilization baskets) along with the monthly maintenance costs, assuming a renewal plan over seven years. The number of surgeries performed annually on the Xi system ranges between 350 and 400 (385 in 2021), with the average annual number of procedures estimated at 380.

### Statistical analysis

Quantitative variables are reported as median (range) values. The non-parametric Kruskal–Wallis test was used to compare sample distributions between the two groups (2018 and 2021). Pairwise cost comparisons were done using the non-parametric Mann–Whitney two-sample test. Qualitative variables are described by the number and frequency of observations for each of the outcomes. The χ2 test was used for comparison of the proportions. Analyses were carried out using SPSS.

## Results

### Patient characteristics

Table 1 summarizes the characteristics of all patients who underwent hepatectomy in 2018 and 2021. The WHO performance status index showed no significant difference between the two years (*p* = 0.34). The average age of patients in 2018 was 63 compared to 62.4 in 2021 (*p* = 0.98). The sex ratio was similar, with a majority of men in both years (*p* = 0.12). The average preoperative BMI was 29.9 in 2018 and 27.4 in 2021 (*p* = 0.05). There was no significant difference in the number of cirrhotic patients: 13 out of 40 in 2018 and 26 out of 71 in 2021 (*p* = 0.66). No Child B patients were recorded. MELD scores were below 10.

**Table 1:** Characteristics of all patients who underwent hepatectomy in 2018 and 2021

Patient characteristics	2018 (*n* = 40)	2021 (*n* = 71)	*p*
Mean age (years)	63	62.4	0.98
Sex			0.34
Female	9	22	
Male	31	49	
BMI (Kg/m^2^)	29.9	27.4	0.05
WHO			0.34
≤ 2	31	49	
> 2	9	22	
Cirrhosis	13	26	0.66
Etiology			0.28
Hepatocarcinoma	18	32	
Cholangiocarcinoma	8	7	
Metastasis	10	17	
Benign	4	15	
Mean lesion size (mm)	42.1	44.7	0.68
Procedure			< 0.001
Open	23	18	
Mini-invasive (lap/robot)	17	53 (10/43)	
Hepatectomy			0.49
Minor	28	54	
Major	12	17	
IWATE score			
Mini-invasive	5.2	5.9	0.38
Mean operating time (minutes)	196	167	0.09

The most common oncologic indication was hepatocellular carcinoma, with 18 cases in 2018 and 32 cases in 2021. This was followed by cholangiocarcinoma and metastases. Indications for hepatectomy in the context of benign pathology were more limited, with 4 cases in 2018 and 15 cases in 2021. Lesion sizes were similar between the two years: 42.1 mm in 2018 and 44.7 mm in 2021 (*p* = 0.68).

### Intraoperative findings

The proportion of minimally invasive surgery increased over time, representing 47.5% of liver surgeries in 2018 (19/40) and 75% in 2021 (53/71) (*p* < 0.01), with 10 laparoscopic and 43 robotic resections. Among the 43 robotic resections, three were converted to open surgery due to bleeding or difficulties in exposure.

Most patients underwent minor hepatectomy: 28 in 2018 and 54 in 2021. In 2018, 12 lesions (30%) were localized in the postero-superior segments (VII–VIII). Of these, seven were wedge resections (not segmentectomies), and the remainder were major resections (right, extended right, and central hepatectomy). More than half of these challenging resections were performed via laparotomy (7/13). The proportion of postero-superior lesions remained constant in 2021 (24/71, 33%). Laparotomy was performed in only 7/24 cases (*p* = 0.17). Half of the resections were anatomic, including five anatomic segmentectomies.

The global IWATE difficulty score was not significantly different between 2018 and 2021, but there was a trend toward increased complexity in minimally invasive procedures (5.2 vs. 5.9). Operative time showed a non-statistical decrease. Four of the 19 minimally invasive resections in 2018 were IWATE grade high or expert (21%), compared to 19/53 (35.8%) in 2021. This indicates that both the number of procedures and their complexity have increased.

### Post-operative course

The proportion of laparotomy procedures decreased from 53 to 25%. Meanwhile, the overall morbidity rate dropped from 32% in 2018 to 18.3% in 2021. The severe morbidity rate also decreased, from 20 to 8.5%. The complication rate in the open surgery group remained constant between 2018 and 2021, as did the severe complication rate (27.3% vs. 33%).

The average overall length of hospital stay in the laparotomy group improved slightly, from 7.7 days in 2018 to 7 days in 2021. However, the shift to a minimally invasive approach had a more significant impact. The mean overall length of hospital stay decreased from 8.6 to 5.1 days, representing a reduction of 3.5 days on average. In 2021, among patients with major complications, those who underwent robotic surgery had shorter stays in a reinforced care unit (4.6 days) compared to those who underwent open surgery (8.2 days).

### Cost analysis

**Fig. 1:**
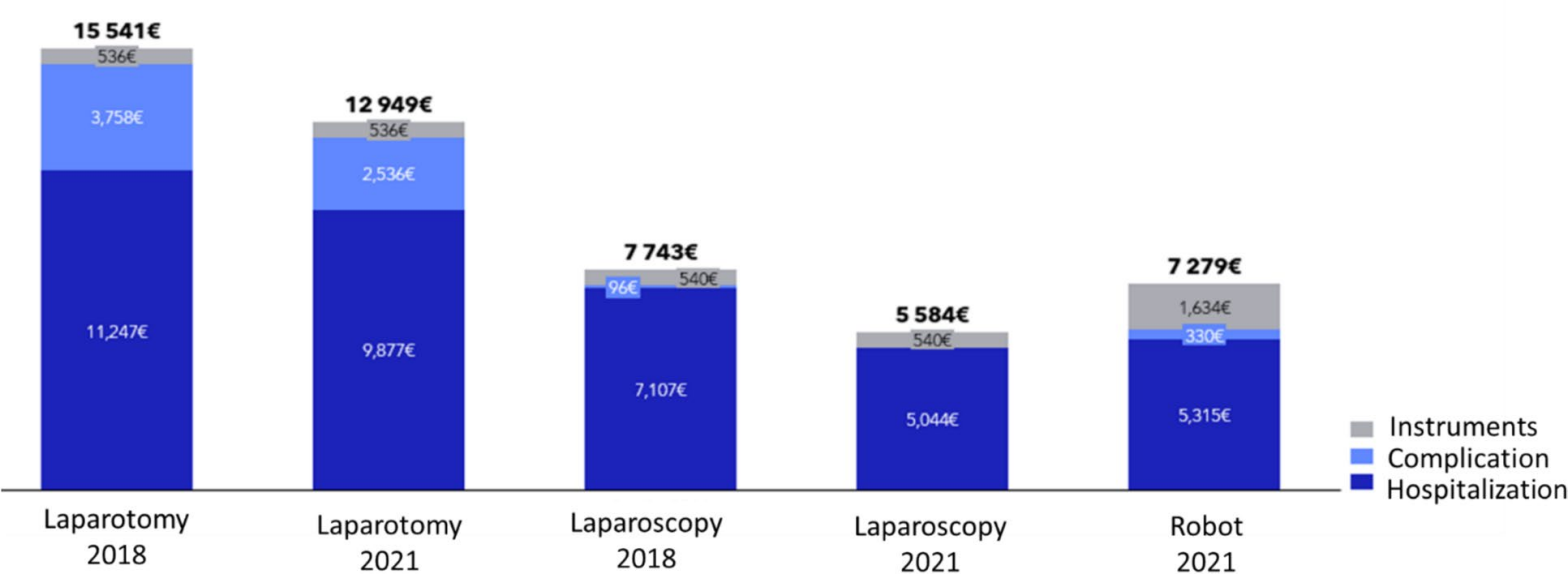
Mean cost per procedure through years

The mean cost per procedure is summarized in Fig. 1. The cost per procedure decreased regardless of the technique employed. However, the robotic approach had higher material costs due to the utilization of disposable instruments compared to the open approach. These additional material costs are associated with instruments with a restricted operational lifespan.

In 2021, the costs associated with managing complications were higher for laparotomy (€2,536) compared to robotic surgery (€330). This difference partially explains the cost variations, but the primary factor remains the effect of reduced hospitalization length. When direct and indirect costs are considered, laparotomy is more expensive than robotic surgery, with a cost ratio of approximately 2:1. The increased use of minimally invasive surgery between 2018 and 2021 corresponded to a reduction in average costs per patient.

**Fig. 2:**
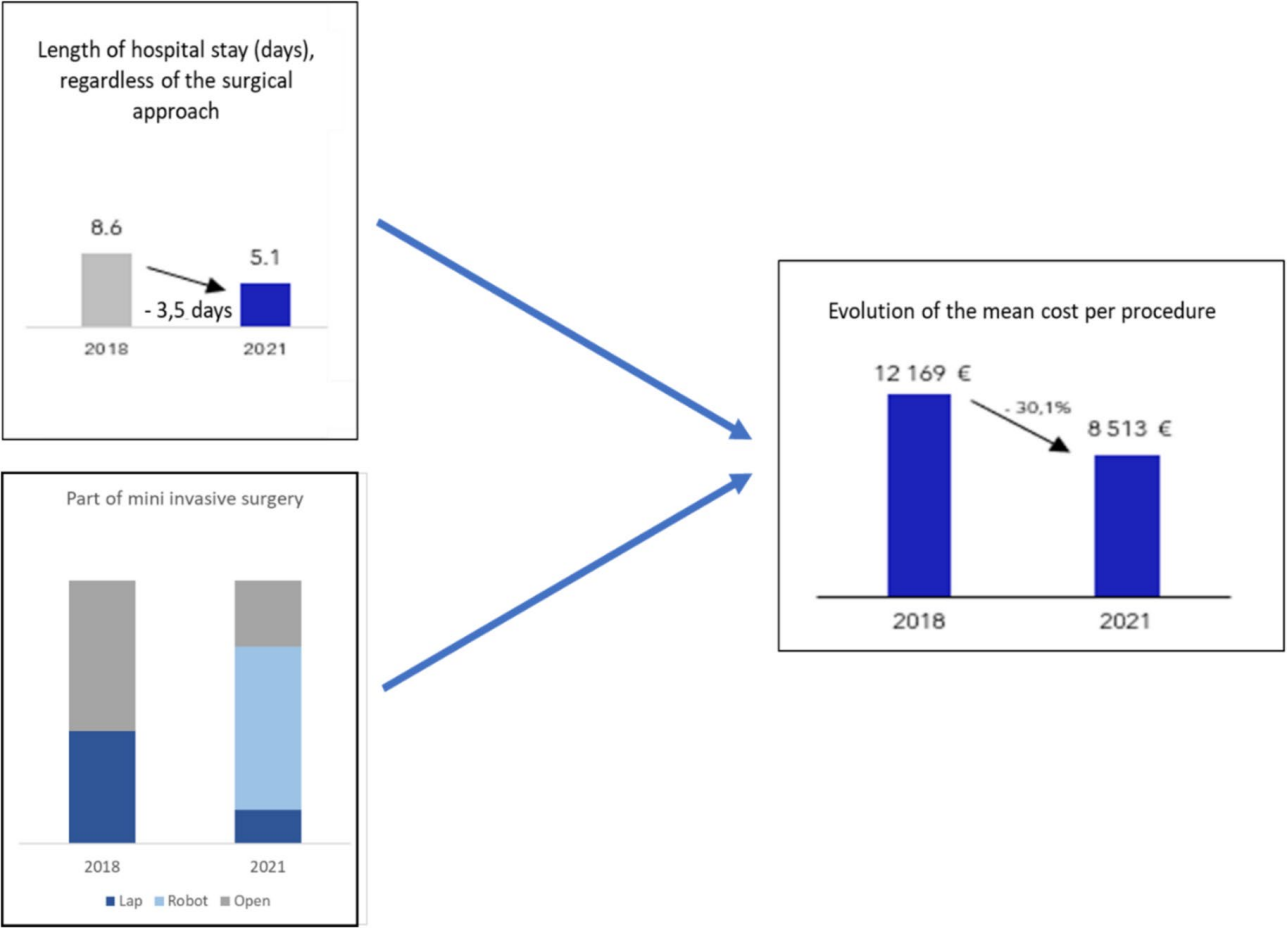
The increase in the proportion of minimally invasive surgery, along with the reduction in mean hospital stay between 2018 and 2021, was associated with a decrease in the mean cost per hepatectomy

Globally, the financial costs associated with hepatectomy decreased from €12,169 in 2018 to €8,513 in 2021. This reduction is partially attributed to the introduction and advancement of robot-assisted liver surgery (Fig. 2). The total savings for the 71 patients in the 2021 cohort amounted to €259,576. When calculating the total cost of the 71 hepatectomies performed in 2021, based on the distribution of open (57%) versus minimally invasive (43%) surgeries from 2018, a cost difference exceeding €100,000 persists, favoring the use of robotic assistance.

The annual operating cost of the robot, including the purchase of the robot, seven additional optics and 30 baskets adapted to the size of the instruments, is approximately €322,000, or just under €850 per operation. It should be noted that the cost of purchasing a new STERRAD low-temperature hydrogen peroxide medical sterilizer for the optics is not included in the calculation, as this platform is being made available to other centers in the region, thereby becoming a source of revenue.

## Discussion

This study highlights that the use of robotic-assisted surgery by an experienced hepatobiliary surgeon does not result in increased costs per procedure despite existing discrepancies in the literature regarding the financial impact of new surgical technologies. The adoption of advanced technologies in medicine is often weighed against the expected benefits for patients, including safety and outcomes, while considering any potential additional costs. Our findings show that robotic liver surgery, when performed by a skilled surgeon, is cost-effective due to reduced indirect costs, without compromising patient outcomes or safety.

Robotic surgery first gained prominence in thoracic, urology and gynecology, where significant cost savings were demonstrated for procedures, such as lobectomy [24], prostatectomy [25] and hysterectomy [5]. For instance, robotic-assisted partial nephrectomy yielded savings of €920 for inpatient procedures and €2,150 for outpatient surgeries [5]. Similarly, in gynecology, robotic hysterectomy was found to be more cost-effective [5]. However, to our knowledge, no studies have explored the financial implications of robotic liver surgery, particularly in the context of a surgeon’s growing expertise. This real-world analysis demonstrates that robot-assisted liver surgery reduces hospital costs primarily through shorter hospital stays and fewer complications, rather than incurring additional expenses [26].

The acquisition of expertise in robotic surgery enables the performance of increasingly complex procedures, such as posterior segment hepatectomy, which were traditionally done via open surgery due to the limitations of laparoscopic techniques [27]. This transition is not applicable to all cases, and we observe the complementary nature of the different surgical approaches, which align at the end of the learning curve. The increase in minimally invasive procedures correlates with a reduction in costs compared to open surgery, particularly when complex cases are handled robotically. When comparing robotic surgery to laparoscopy, the ability to perform difficult tasks like posterior segment resections becomes a significant advantage, alongside improved patient outcomes, as shown by Sijberden et al. [28].

The surgeon’s learning curve for robotic liver surgery took place between 2019 and 2020. During this period, the surgeon performed approximately 50 robotic procedures, reaching the threshold of experience defined in the literature as the point at which a surgeon is considered proficient in robotic surgery [29, 30]. This allowed for a direct comparison between laparotomy and robotic surgery, with variables limited to formal contraindications to robotics and expected patient benefits. By eliminating the bias associated with the surgeon’s lack of experience, we were able to conduct a precise evaluation of the costs generated by robotic surgery without technical limitations. The increasing feasibility of performing complex procedures with robotic assistance after the learning curve has led to a more limited use of laparoscopy for simpler cases. In 2021, only patients with low or medium IWATE severity grades were treated laparoscopically (none with severity index III–IV), while 21% of robotic-assisted patients had a severity index of III–IV. This discrepancy helps explain the additional 330€ costs associated with robotic surgery.

Additionally, the integration of robotic surgery within the medical and paramedical teams has improved logistical efficiency. The smooth coordination of patient preparation, robot setup, and recovery has reduced the time required for these processes. As the teams gained familiarity with the technology, the setup time became quicker, and fewer surgical instruments were required, further contributing to cost savings. This optimization is both technical—due to the surgeon’s completed learning curve—and logistical due to the medical team’s experience, ultimately making patient hospitalization more efficient and financially viable for the hospital like in urology [31].

Compared to other surgical technologies, the robot is characterized by a high initial investment and significant instrumentation costs, as instruments can only be temporarily reused. However, the findings of this study show that when considering the broader indirect benefits, the robot provides substantial savings that outweigh these fixed costs. Taking cost evolution into account, the estimated savings for the 71 patients who underwent surgery in 2021 amount to approximately €100,000. This alone covers about a third of the annual investment and maintenance costs, even though the study includes only 41 of the 385 patients who underwent robotic surgery that year. Nonetheless, the initial financial outlay remains significant, and the example of the sterilizer in our center could serve as a model for resource pooling and funding generation. Similarly, exploring equipment leasing options to alleviate the financial impact of the initial investment is a relevant consideration.

These findings underscore the financial sustainability of robotic surgery in hepatobiliary procedures once the surgeon has achieved proficiency. While musculoskeletal issues arising from laparotomy and laparoscopy are not directly factored into cost analyses, they are significant for the surgeon’s long-term health. The robot’s ergonomic benefits [32] contribute to the sustainability of the surgeon’s career, which is an important factor in the overall cost-effectiveness of this technology.

The implementation of robotic surgery also enhances patient care, providing additional benefits beyond cost savings. The increased demand for robotic surgery can accommodate a larger patient population [8], which not only improves individual outcomes but also fosters the development of robotic surgery at a national and global level. The experience gained by the center enhances its reputation, attracting both patients and professionals. Furthermore, the center’s success in robotic surgery boosts its standing in the global medical community through publications and shared expertise.

Several limitations should be acknowledged. The retrospective design of this study introduces potential selection bias, a common issue in similar research. Furthermore, the study’s single-center setting may limit the generalizability of the findings. Additionally, changes in clinical practices, particularly with regard to postoperative rehabilitation, likely contributed to part of the observed reduction in length of stay between 2018 and 2021. However, we believe that the significant increase in the proportion of minimally invasive surgeries has fostered an overall trend toward accelerated rehabilitation, creating a positive feedback loop that leads to shorter hospital stays, even for patients undergoing open surgery. Another limitation of this study is that the length of hospital stay may not accurately reflect the quality of the postoperative period, as it is influenced by factors unrelated to recovery, such as the patient’s proximity to home, age, and the availability of family support. LOS is influenced by the necessity for postoperative monitoring, particularly in complex cases [33], even those which are performed via a minimally invasive approach. In the present study, a decrease in the LOS was observed as the complexity of cases increased, thereby underlining the positive impact of minimally invasive surgery on current practices.

Future studies should aim to incorporate multicenter data and evaluate long-term outcomes, including patient-reported quality of life metrics but also surgeon-related metrics, to provide a more comprehensive assessment.

Moreover, the environmental impact of robotic surgery should be explored. The use of disposable instruments, while critical for maintaining sterility and functionality, contributes to medical waste. Balancing the benefits of robotic technology with sustainable practices represents an important area for future research.

Finally, advancements in robotic technology, such as the development of new platforms and instruments, may further enhance the cost-effectiveness and accessibility of robotic surgery. Continuous innovation and competition in the field are likely to drive down costs and improve patient outcomes, making robotic surgery an integral part of modern hepatobiliary care.

## Conclusion

This study demonstrates that robot-assisted hepatectomy, when performed by an experienced hepatobiliary surgeon, offers significant clinical and financial benefits. The use of robotic technology does not lead to increased costs per procedure, despite the additional material expenses associated with the robotic platform. Instead, it results in cost savings primarily through reduced hospitalization times, fewer complications, and improved surgical outcomes, particularly for complex procedures such as posterior segment hepatectomies.

In conclusion, the integration of robotic surgery in hepatobiliary procedures is a promising step toward more efficient, cost-effective, and patient-centered care. Further studies exploring long-term outcomes and scalability are necessary to fully establish the broader economic impact of robotic surgery in this field.

## Data Availability

The data that support the findings of this study are not openly available due to reasons of sensitivity and are available from the corresponding author upon reasonable request. Data are located in controlled access data storage at Lille University Hospital.
